# AUY922 Effectively Overcomes MET- and AXL-Mediated Resistance to EGFR-TKI in Lung Cancer Cells

**DOI:** 10.1371/journal.pone.0119832

**Published:** 2015-03-17

**Authors:** Yun Jung Choi, Seon Ye Kim, Kwang Sup So, In-Jeoung Baek, Woo Sung Kim, Se Hoon Choi, Jae Cheol Lee, Trever G. Bivona, Jin Kyung Rho, Chang-Min Choi

**Affiliations:** 1 Department of Pulmonology and Critical Care Medicine, Asan Medical Center, College of Medicine, University of Ulsan, Seoul, Korea; 2 Asan Institute for Life Sciences, Asan Medical Center, College of Medicine, University of Ulsan, Seoul, Korea; 3 Thoracic and Cardiovascular Surgery, Asan Medical Center, College of Medicine, University of Ulsan, Seoul, Korea; 4 Department of Oncology, Asan Medical Center, College of Medicine, University of Ulsan, Seoul, Korea; 5 Division of Hematology/Oncology, Helen Diller Comprehensive Cancer Center, University of California San Francisco, San Francisco, California, United States of America; University of Navarra, SPAIN

## Abstract

The activation of bypass signals, such as MET and AXL, has been identified as a possible mechanism of EGFR-TKI resistance. Because various oncoproteins depend on HSP90 for maturation and stability, we investigated the effects of AUY922, a newly developed non-geldanamycin class HSP90 inhibitor, in lung cancer cells with MET- and AXL-mediated resistance. We established resistant cell lines with HCC827 cells harboring an exon 19-deletion mutation in of the *EGFR* gene via long-term exposure to increasing concentrations of gefitinib and erlotinib (HCC827/GR and HCC827/ER, respectively). HCC827/GR resistance was mediated by MET activation, whereas AXL activation caused resistance in HCC827/ER cells. AUY922 treatment effectively suppressed proliferation and induced cell death in both resistant cell lines. Accordingly, the downregulation of EGFR, MET, and AXL led to decreased Akt activation. The inhibitory effects of AUY922 on each receptor were confirmed in gene-transfected LK2 cells. AUY922 also effectively controlled tumor growth in xenograft mouse models containing HCC827/GR and HCC827/ER cells. In addition, AUY922 reduced invasion and migration by both types of resistant cells. Our study findings thus show that AUY922 is a promising therapeutic option for MET- and AXL-mediated resistance to EGFR-TKI in lung cancer.

## Introduction

Epidermal growth factor receptor-tyrosine kinase inhibitor (EGFR-TKI) is one of the most successful targeting agents used to treat cancer. However, the development of resistance, despite good initial responses, in patients with EGFR-mutant lung cancer is inevitable [1,2]. Although almost half of all TKI resistance is caused by a secondary T790M mutation [3,4], the activation of bypass signals such as MET or AXL could also contribute to the acquisition of resistance [5,6].

MET gene amplification causes HCC827 cells harboring the sensitizing EGFR mutation to become resistant to gefitinib via ErbB3-dependent activation of the phosphoinositide 3-kinase/Akt (PI3K) pathway [5]. Initial studies reported that approximately 20% of patients with acquired resistance to EGFR-TKIs showed *MET* gene amplification with or without T790M [5,7], while a recent study on the frequency of resistance mechanisms revealed that *MET* amplification developed in approximately 5% of patients after resistance [8]. Combination treatments with MET and EGFR inhibitors could abrogate the activation of downstream signals, thereby overcoming acquired resistance to EGFR inhibitors [5,9]. Several MET tyrosine kinase inhibitors and MET-blocking monoclonal antibodies, including SU11274, ARQ197 and onartuzumab, are in clinical trials [10].

Recently, three independent study groups reported that AXL, which is included in the TAM (Tyro-Axl-Mer) receptor tyrosine kinase (RTK) family, could be a cause of EGFR-TKI resistance in preclinical models [6]. Although approximately 20% of patients demonstrated AXL over-expression after developing resistance in that study, the exact proportion among patients with acquired resistance and the treatments that could be used to overcome the effects of targeting AXL in clinical settings remain to be determined [11].

Heat shock protein 90 (HSP90) plays a critical role in maintaining cellular protein homeostasis by affecting protein maturation and stability [12]. Because various oncoproteins depend on its proper function, HSP90 has been recognized as an attractive therapeutic target [13]. Clinical trials targeting mutant EGFR, including the T790M mutant with HSP90 inhibitors, are in progress. Some preclinical studies have reported promising results [14–16]. However, there are insufficient data on how MET- or AXL-mediated resistance to EGFR-TKI in lung cancer could be overcome by inhibiting HSP90. In our present study, we investigate the efficacy of AUY922, a non-geldanamycin class HSP90 inhibitor of MET- and AXL-mediated resistant cell lines and animal models.

## Materials and Methods

### Cell culture and reagents

HCC827 cells were obtained from the American Type Culture Collection (Rockville, MD). Gefitinib- and erlotinib-resistant cell lines (HCC827/GR and HCC827/ER, respectively) were established as part of a previous study [17]. Cells were cultured in RPMI 1640 (Invitrogen, Carlsbad, CA) containing 10% fetal bovine serum (FBS), 100 U/mL penicillin, and 100 μg/mL streptomycin (Invitrogen) at 37°C in an atmosphere of 5% CO_2_. AUY922 was purchased from Selleck Chemicals (Houston, TX).

### Cell viability assays

Cell viability was assessed using the MTT assay. Briefly, cells in the logarithmic growth phase were harvested, seeded onto 96-well plates, and cultured overnight. Cells were exposed to various doses of AUY922 in medium containing 1% FBS. After 72 hours, the MTT assay was performed as described by Carmichael et al. [18]. To validate the anticancer effects of AUY922, cells were treated with the indicated doses of AUY922 for 72 hours and the attached cells were stained with a 0.2% trypan blue solution containing 50% methanol. Cell viability was determined using an ADAM-MC automatic cell counter (NanoEnTek, Seoul, Korea) in accordiance with the manufacturer’s instructions. Results are representative of at least three independent experiments, and the error bars indicate the standard deviation (SD).

### Western blot analysis

For western blotting, proteins were separated on SDS-polyacrylamide gels and electrotransferred to Immobilon-P membranes (Millipore, Bedford, MA). Antibodies specific for p-EGFR (Tyr1173), EGFR, AXL, MET, Akt, p-Erk, Erk, Myc, and β-actin were obtained from Santa Cruz Biotechnology (Santa Cruz, CA), and antibodies for p-MET (Tyr1234/1235), p-AXL (Tyr702), p-Akt, PARP and caspase-3were obtained from Cell Signaling Technology (Beverly, MA). Proteins were detected using an enhanced chemiluminescence western blotting kit (Amersham Biosciences, NJ)in accordance with the manufacturer’s instructions.

### Transient transfection

pcDNA3/EGFR(WT)and pcDNA3/EGFR (delE746-A750) were provided by Dr. T.Y. Kim (College of Medicine Seoul National University, Seoul, South Korea). pCMV6-Entry/MET was purchased from OriGene (Rockville, MD). pcDNA3.1/AXL (WT) was constructed as part of a previous study [6]. Transfections were performed using Lipofectamine 2000 (Invitrogen) in accordance with the manufacturer’s instructions.

### Cell cycle analysis

Cells were seeded onto 6-well plates and treated for the indicated times with 1 μmol/L AUY922. Both adherent and floating cells were collected for analysis. Cells were fixed in 70% ice-cold ethanol for 1 hour and incubated in 50 μg/mL RNase A and 25 μg/mL propidium iodide (PI) for 30 minutes at 37°C. Quantitative analysis of the cell cycle distribution was performed using FASCalibur flow cytometry (Becton Dickinson, Franklin Lakes, NJ).

### *In vivo* study

Female severe combined immunodeficiency (SCID) mice (18–20 g; 5 weeks of age; 5 mice/group) were purchased from Charles River Laboratories. All experimental procedures were conducted following a protocol approved by the Institutional Animal Care and Use Committee of Asan Institute for Life Sciences (2013–01–066). Tumors were grown by implanting cells (1 × 10^6^ HCC827/GR cells or 5 × 10^6^ HCC827/ER cells) in Matrigel (BD Biosciences) and subsequently into the mouse flanks. Treatment with the vehicle control commenced when the tumors reached a volume of 50–100 mm^3^ (20 mg/kg AUY922 via intra-peritoneal injection; 5 days/week). Treatment was stopped on the indicated day, and mice received follow-up examinations to document tumor recurrence. To measure tumor size, the length (*L*) and width (*W*) of each tumor was measured using calipers, and tumor volume (TV) was calculated as TV = (*L*×*W* ^2^)/2. Immunohistochemical staining was performed using a specific primary antibody (Ki-67; DakoCytomation, Los Angeles, CA), the EnVision Plus staining kit (DakoCytomation), and the APO-Direct terminal deoxynucleotidyl transferase-mediated dUTP nick end labeling (TUNEL) assay kit (Millipore) as directed by the supplier’s instructions. Quantitative analysis of each stained section was performed by counting all immunopositive cells in 5 arbitrarily selected fields (×400 magnification).

### Invasion and migration assays

All cell migration and invasion assays were performed according to a previously described method [19]. Results are representative of at least three independent experiments, and the error bars indicate SDs.

## Results

### AUY922 effectively inhibits the proliferation of gefitinib/erlotinib-resistant cells

**Fig 1:**
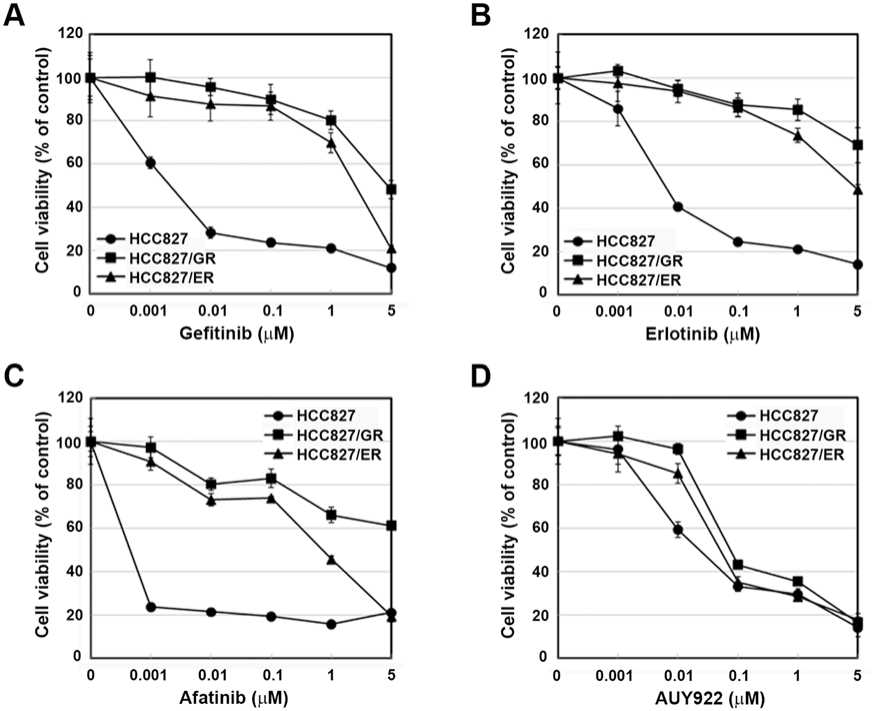
EGFR-TKI and AUY922 cytotoxicity in parental HCC827 and resistant cell lines. Cells were treated with the indicated doses of gefitinib, erlotinib, afatinib, or AUY922 for 72 hours in medium containing 1% FBS. Cell viability was determined using the MTT assay.

**Fig 2:**
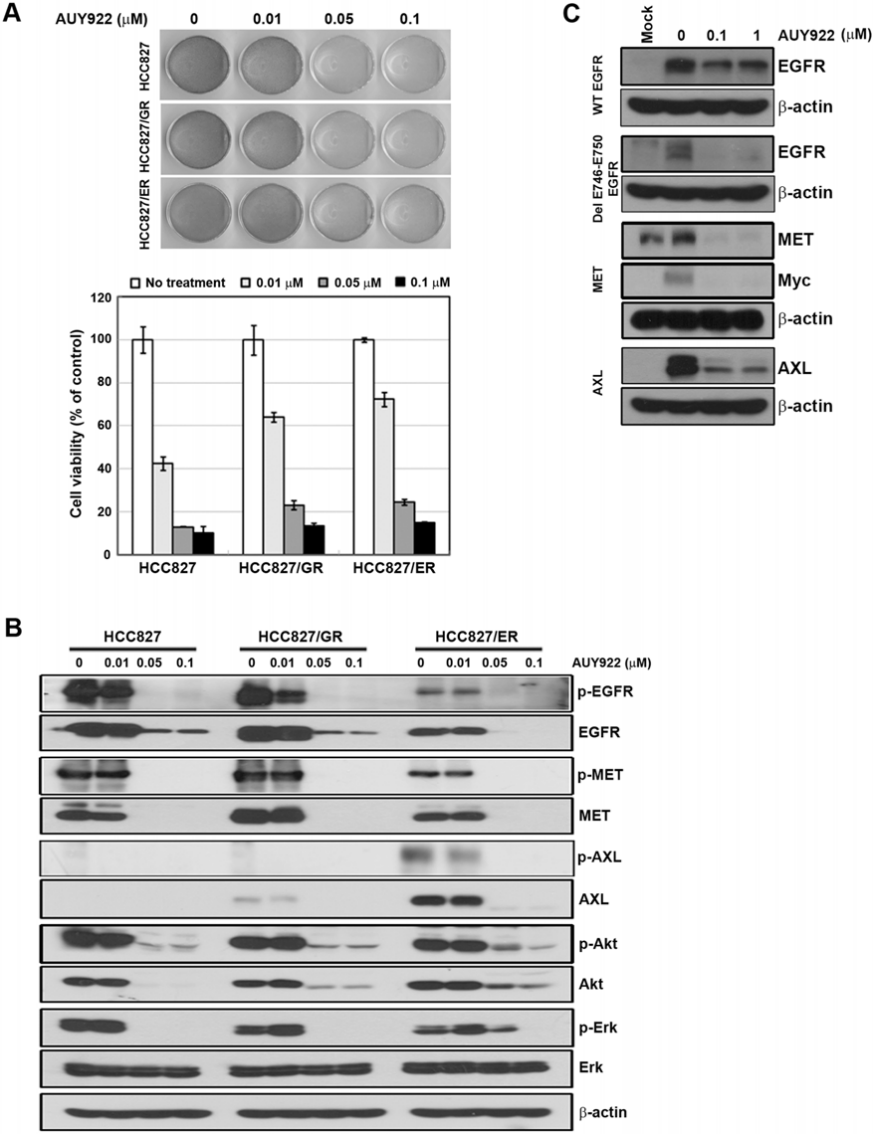
Suppression of MET and AXL by AUY922 in resistant cell lines. **A**, Cells were treated with the indicated doses of AUY922 for 72 hours in medium containing 1% FBS. Attached cells were stained with trypan blue solution (top). Cell viability based on cell counting is also shown (bottom). Bars represent the mean ±SD of three wells. **B**, Cells treated with AUY922, similar to panel A. After 48 hours, cells were harvested and EGFR-related signaling molecules were evaluated using western blotting. **C**, LK2 cells were treated with vector-containing wild-type EGFR, del E746-E750, MET, or AXL and the indicated doses of AUY922 for 12 hours.

Gefitinib/erlotinib-resistant sublines derived from the parental, sensitive HCC827 cell line were established as part of a previous study[17]. Cells were treated with AUY922 in a dose-dependent manner to determine if it inhibits cell growth in gefitinib/erlotinib-resistant cells. As shown in Fig. 1, both resistant cell lines demonstrated cross-resistance to gefitinib or erlotinib, respectively, and were also resistant to afatinib. Nevertheless, AUY922 treatment effectively suppressed the proliferation of the parental line and both resistant cell lines (Fig. 1D). The growth-inhibiting effects of AUY922 were observed even when the drug concentration was low (Fig. 2A).

We have previously demonstrated that the resistance of HCC827/GR and HCC827/ER cells is mediated by MET or AXL activation, respectively [6,17]. Previous reports have also indicated that a large number of HSP90 client proteins have crucial roles in cancer progression, and that the inhibition of HSP90 degrades client proteins [13,20,21]. Thus, we investigated whether AUY922 affects the stability of MET or AXL. The inhibition of HSP90 by AUY922 was found to lead to the significant degradation of EGFR, MET, and AXL in a dose- (Fig. 2B) and time-dependent manner (S1 Fig.). Consistent with this, AUY922 treatment also decreased the level of artificially induced proteins in LK2 cells that were transfected with genes such as *EGFR* (WT), *EGFR* (Del E746-E750), *MET*, and *AXL* (Fig. 2C).

**Fig 3:**
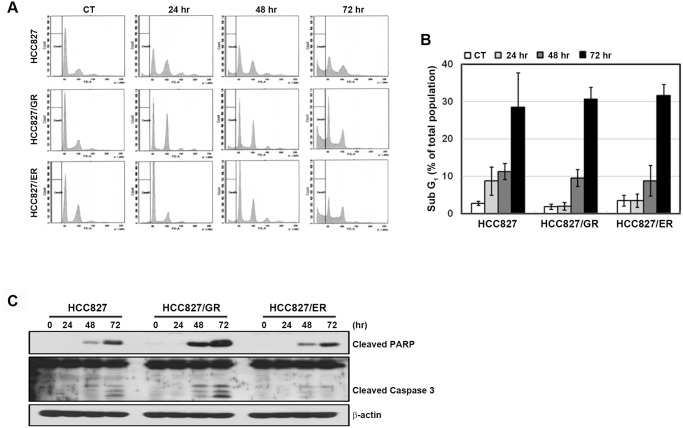
Induction of cell death in response to AUY922. **A** and **B**, Cells were treated with 0.1 μmol/L AUY922 for 24–72 hours. To analyze the cell cycle distribution, the harvested cells were analyzed by flow cytometry. **C**, Cell lysates were analyzed using western blotting. The indicated antibodies were used to evaluate apoptotic cell death.

Many HSP90 inhibitors induce cell cycle arrest and apoptosis in various cancer cells [22–26]. To further confirm the effects of AUY922, we examined cell cycle changes in PI-stained cells using FACS analysis. As shown in Fig. 3A, AUY922 treatment increased the number of cells in G2/M phase and induced sub-G1 (i.e., cell death) after 48 hours. Consistent with these results, time-dependent PARP and caspase-3 cleavage was also observed (Fig. 3C). These data indicated that the antitumor effects of AUY922 on parental and resistant cells may result from the induction of cell cycle arrest and cell death.

### AUY922 overcomes acquired resistance to gefitinib/erlotinib in xenograft models

**Fig 4:**
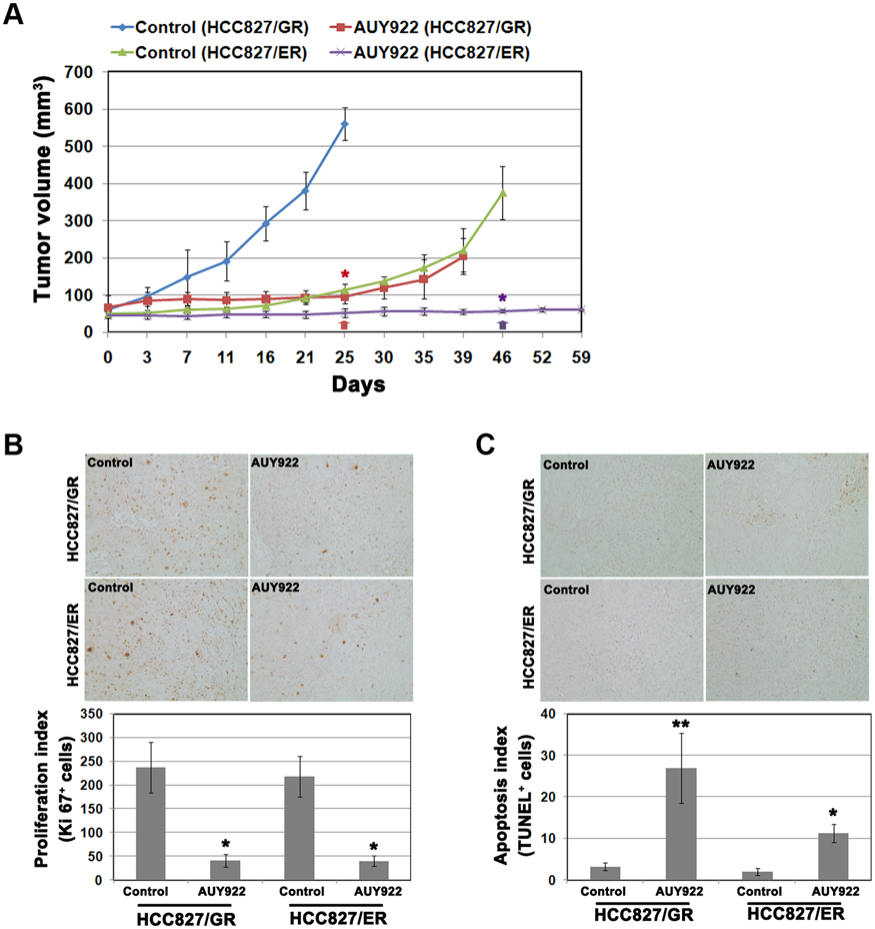
Efficacy of AUY922 in HCC827/GR and HCC827/ER tumor xenograft models. **A**, SCID mice with established HCC827/GR and HCC827/ER tumor cell xenografts were treated with AUY922. Tumor size was measured on the indicated days, and tumor volume was calculated. Arrows mean last day of drug treatment (red arrow, HCC827/GR; purple arrow, HCC827/ER). Bars represent the mean tumor volume ± SD. **B** and **C**, Immunohistochemical staining for Ki-67 and TUNEL followed by quantitative analysis of proliferation and apoptosis in (B) Ki-67^+^ cells and (C) TUNEL^+^ cells. *p< 0.01 and **p< 0.001 in comparison with the control.

HCC827GRand HCC827/ER tumor xenografts were established in SCID mice to further evaluate the antitumor efficacy of AUY922. The tumor growth rate of HCC827/ER cells was lower than that of HCC827/GR cells. AUY922 treatment resulted in the substantial inhibition of growth in both cells (Fig. 4A). Suppressed tumor growth was maintained in HCC827/ER xenografts through 2 weeks after drug discontinuation, whereas tumors in the HCC827/GR xenograft slowly grew again following drug withdrawal. Immunohistochemical analyses of tumor tissues revealed that cell proliferation had decreased and apoptosis was induced in both tumors by AUY922 treatment (Fig. 4B-C).

### AUY922 decreases cellular mobility

**Fig 5:**
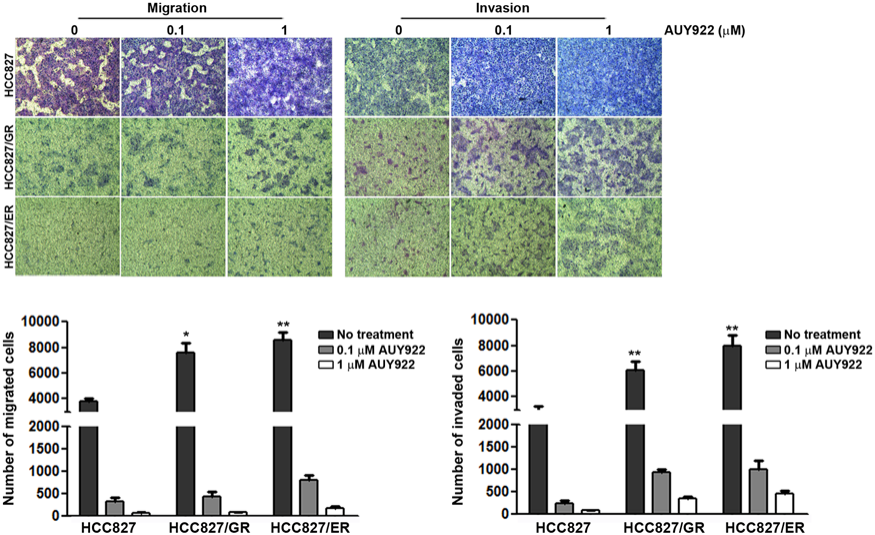
Reduced migratory and invasive capabilities of cancer cells following AUY922 treatment. Cells were seeded onto either collagen or matrigel-coated polycarbonate filters to determine their migratory and invasive potentials, respectively. Cells were incubated in modified Boyden chambers with or without the indicated doses of AUY922 for 24 hours, and the cells that penetrated the filter were stained and counted using a light microscope. Bars represent the mean ± SD of three wells. *p< 0.01 and **p< 0.001 in comparison with HCC827 cells.

The induction of AXL or MET is associated with increased cellular mobility in cancers [6,10,27–29].We therefore investigated changes in cellular mobility in both the parental and gefitinib/erlotinib-resistant cells. As expected, the migratory and invasive abilities in both resistant cell types dramatically increased in comparison with parental cells (Fig. 5). AUY922 treatment resulted in reduced migratory and invasive capabilities in both resistant and parental cells.

## Discussion

Since mutant EGFR variants, including T790M, were first shown to be associated with HSP90 chaperone proteins [16], multiple similar lines of evidence have accumulated. T790M-expressing cells that are resistant even to irreversible EGFR-TKIs are sensitive to HSP90 inhibition. HSP90 inhibition leads to the regression of EGFR *L858R*-driven murine lung adenocarcinoma, regardless of the presence of T790M[15]. Bao et al. have also reported that targeting HSP90 with CUDC-305, a HSP90 inhibitor, can overcome erlotinib-resistance in non-small cell lung cancer [30]. Furthermore, 17-DMAG reduces the growth of lung cancer cells with mutant EGFR, irrespective of the presence of a T790M mutation in mice xenograft models [14]. Currently, over a dozen HSP90 inhibitors are enrolled in clinical trials, some of which are investigating T790M-mediated resistance to EGFR-TKIs [20].

Several earlier studies have revealed that MET is a HSP90 client protein and is subject to degradation upon HSP90 inhibition. SNX-2112, a novel small molecule inhibitor of HSP90, reduces EGF cross-talk and activation of the c-Met receptor via c-Met degradation in lung cancer cells [31]. Ueno et al. have shown that AUY922 is effective against most NSCLC cell lines, regardless of known molecular alterations. AUY922 treatment was found to induce the significant depletion of client proteins, such as EGFR, MET, and Akt, thereby increasing apoptosis [32]. Although these studies have demonstrated the capability of HSP90 inhibitors to down-regulate MET, they did not evaluate MET-mediated resistance to EGFR-TKI in lung cancer. Accordingly, Koizumi et al. first demonstrated the efficacy of HSP90 inhibitors to overcome resistance caused by MET signaling [33]. In that study, HGF-gene transfected Ma-1 cells with EGFR *L858R* (Ma-1/HGF) did not respond to erlotinib, whilst 17-DMAG inhibited cell proliferation and induced apoptosis by decreasing the expression of EGFR and MET, even under HGF stimuli. Additional clinically relevant models were studied by Xu et al. [34]. These authors generated transgenic mouse models expressing mutant Del19-T790M or L858R-T790M EGFR (each with concurrent MET over-expression). Combination EGFR and MET inhibition with WZ4002 (a third-generation EGFR-TKI) and crizotinib demonstrated highly efficacious control in these cancer models, but neither of these drugs alone could induce a suppressive response. These authors further found that the effects of 17-DMAG are comparable to crizotinib when combined with WZ4002, thus suggesting that MET inhibition is possible via HSP90. Furthermore, a similar effect was observed in another mouse model bearing gefitinib-resistant MET-amplified HCC827 xenografts. These results are almost in agreement with our current findings in a HCC827/GR model. However, our present results better represent a clinical setting because our HCC827/GR cells were established by continuous long-term drug exposure.

Compared with MET, there are few data on how AXL depends on HSP90 for stability. Recently, Krishnamoorthy et al. reported that 17-AAG induces the down-regulation of endogenous or ectopically expressed AXL protein in thyroid cancer cell lines and in HeLa cells in a time- and dose-dependent manner, leading to the inhibition of AXL-mediated signaling and biological activity [35]. To our knowledge, our current study is the first to demonstrate that HSP90 inhibition by AUY922 can overcome AXL-mediated resistance in lung cancer cell lines and animal models. Taken together therefore, the evidence indicates that because HSP90 inhibition can overcome the acquired resistance to EGFR-TKIs caused by the three major resistance mechanisms (T790M, MET, and AXL), HSP90 inhibition could be a promising option for treating EGFR-TKI resistance.

In present studies, we have shown that AUY922 completely suppressed tumor growth in both cells. However, both resistant cells showed the different pattern in growth rate and regrowth after drug discontinuation. HCC827/GR cells grow faster than HCC827/ER cells under condition of drug-free and they showed regrowth following drug withdrawal. The growth rate of HCC827/ER cells was slower than HCC827/GR cells *in vitro* (S2 Fig.). Although various factors can affect tumor growth in xenograft models, the differences of growth rate may influence tumor growth. Actually, HCC827/ER cells had lower EGFR as well as Akt activity than HCC827/GR cells in basal level (data not shown). There were no differences in apoptosis, necrosis, cell cycle arrest (G2/M arrest, S3 Fig.) and proliferation between these two tumors. Thus, the different response to drug withdrawal remained unclear.

Metastasis depends on multiple processes, such as the detachment of tumor cells from the primary tumor, migration, invasion of the surrounding stroma and blood vessels, and survival and proliferation at distant sites. Hepatocyte growth factor (HGF) is the only known ligand of the MET and it is recognized as a fibroblast-derived factor that promotes epithelial cell detachment and stimulates invasion [36,37]. MET affects cell migration, growth in embryogenesis, and controls growth and metastasis in cancer cells [38]. Accordingly, Wu et al. have demonstrated that LY2801653 (a MET inhibitor) significantly inhibits both primary tumor growth and metastasis to the lymph nodes and chest wall in H441 orthotopic models [39]. AXL is also associated with tumor cell invasion and metastasis and promotes tumor growth in various kinds of cancer. Forced ectopic AXL expression induces the highly invasive characteristics seen in MCF7 breast cancer cells from initial weakly invasive phenotypes, whereas using shRNA treatment to induce AXL knockdown decreases the invasive and migratory capabilities of highly invasive cancer cells [29]. In addition, AXL is needed for MDA-MB-231 breast cancer cells to metastasize to the lungs in orthotopic models [27]. In our current analyses, AUY922 significantly reduced the invasive and migratory capabilities of both tumor cells with MET or AXL activation. Further investigations of the role of HSP90 inhibitors in cancer metastasis are needed.

In summary, AUY922 exerts effective control over malignant phenotypes that are driven by MET and AXL, including drug resistance, in lung cancer.

## Supporting Information

S1 Fig Cells were treated with 0.1 μmol/L AUY922 in a time-dependent manner. Experiments were performed as described in Fig. 2A and B.
(TIF)

S2 Fig Cells (5 X 10^3^) were seeded into 24-well plates with completed media containing 10% FBS. Cell numbers were determined with an ADAM-MC automatic cell counter.
(TIF)

S3 Fig Tumor tissues from each group were homogenized for lysate preparation and analyzed by Western blot to evaluated G2/M arrest. Cyclin B1, p21 and cdc2 antibodies were purchased from Santa Cruz Biotechnology (Santa Cruz, CA), and p-cdc2 (Tyr15) was obtained from Cell Signaling Technology (Beverly, MA).
(TIF)
