# Supplementary figures and images for: AUY922 Effectively Overcomes MET- and AXL-Mediated Resistance to EGFR-TKI in Lung Cancer Cells
Source: PLoS One. 2015 Mar 17;10(3):e0119832. doi: 10.1371/journal.pone.0119832 (PMC4363657; doi:10.1371/journal.pone.0119832)

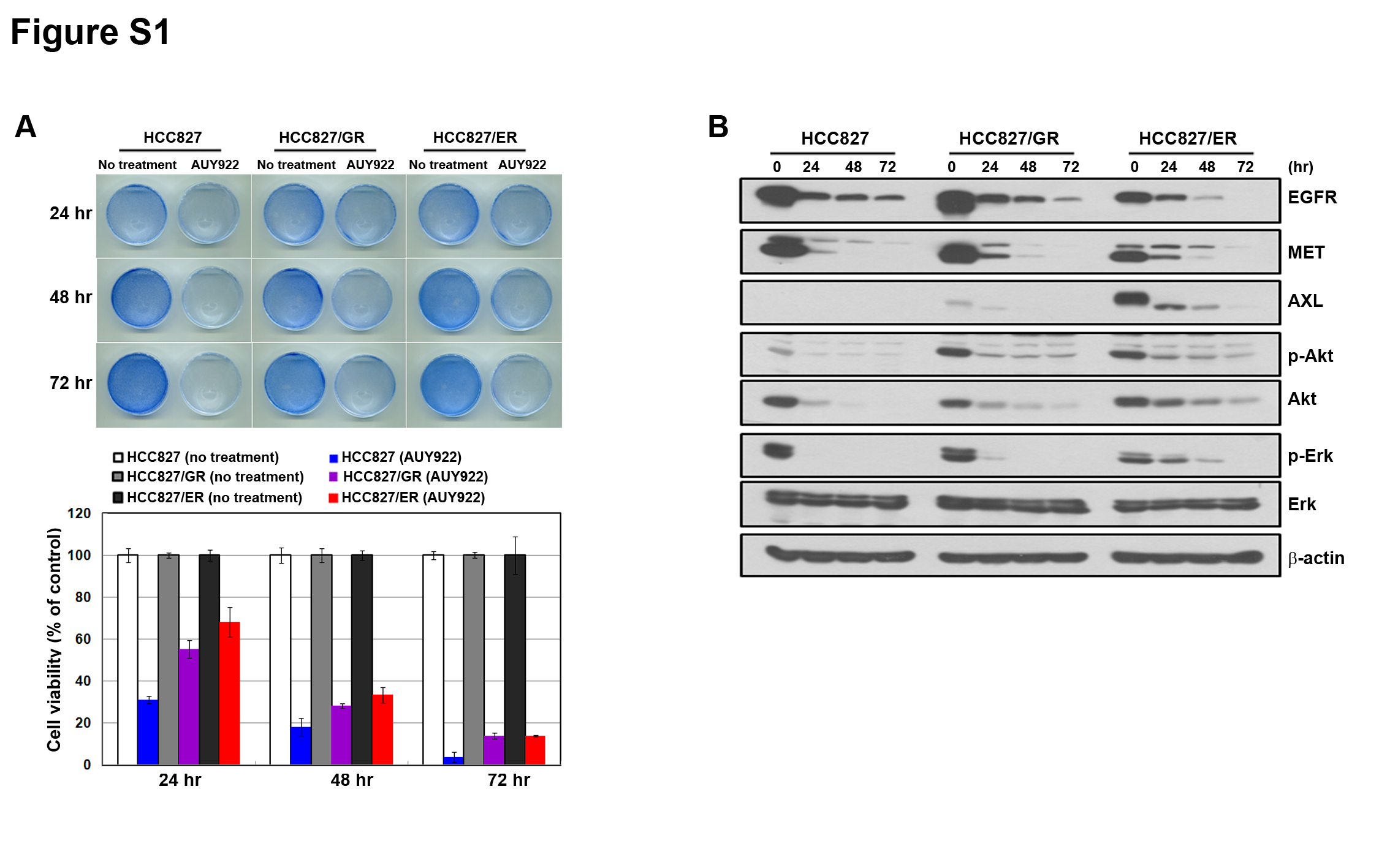

Supplement: S1 Fig — Experiments were performed as described in Fig. 2A and B. (TIF) [file pone.0119832.s001.tif]

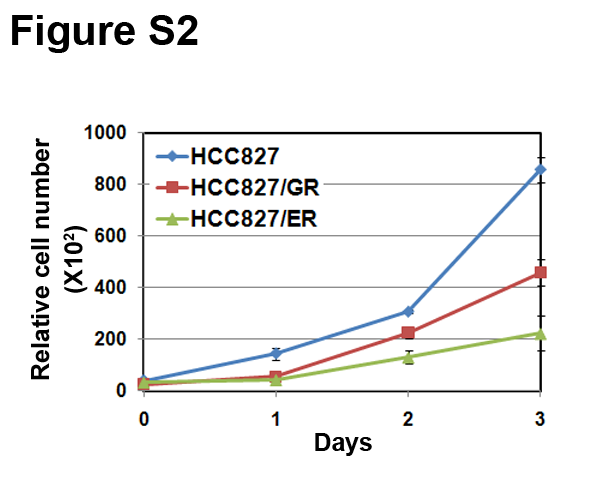

Supplement: S2 Fig — Cell numbers were determined with an ADAM-MC automatic cell counter. (TIF) [file pone.0119832.s002.tif]

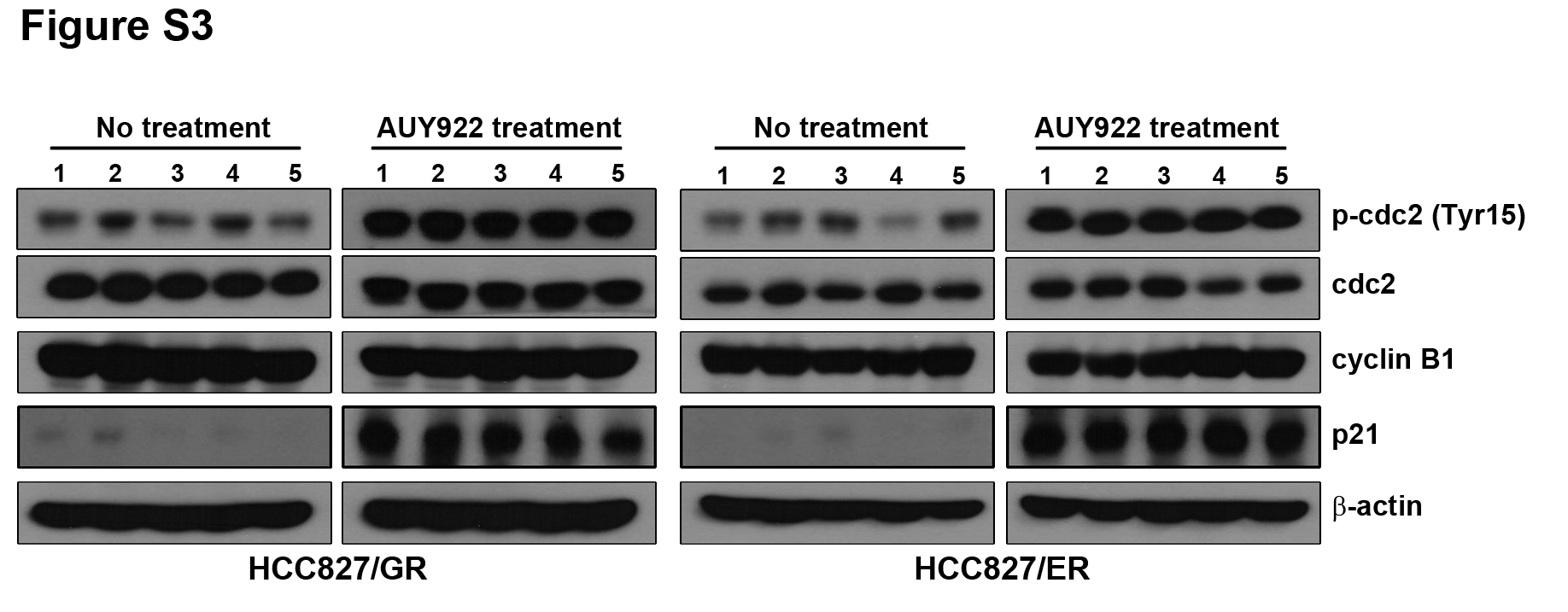

Supplement: S3 Fig — Cyclin B1, p21 and cdc2 antibodies were purchased from Santa Cruz Biotechnology (Santa Cruz, CA), and p-cdc2 (Tyr15) was obtained from Cell Signaling Technology (Beverly, MA). (TIF) [file pone.0119832.s003.tif]
